# Adherence to Non-Invasive Ventilation in Steinert Disease: Clinical and Psychological Insights

**DOI:** 10.3390/brainsci15090968

**Published:** 2025-09-06

**Authors:** Anna Annunziata, Francesca Simioli, Giorgio Emanuele Polistina, Anna Michela Gaeta, Maria Cardone, Camilla Di Somma, Raffaella Manzo, Antonella Marotta, Cecilia Calabrese, Giuseppe Fiorentino

**Affiliations:** 1 Unit of Respiratory Pathophysiology, Monaldi-Cotugno Hospital, 80131 Naples, Italy; annunziatapneumologia@gmail.com (A.A.); francesimioli@gmail.com (F.S.); giorgiopolistina@gmail.com (G.E.P.); cardone.maria@virgilio.it (M.C.); camilladisomma@gmail.com (C.D.S.); raffaellamanzo@gmail.com (R.M.); austen.anto@gmail.com (A.M.); giuseppefiorentino1@gmail.com (G.F.); 2 Department of Respiratory Medicine, Hospital Universitario Severo Ochoa, Av. de Orellana, s/n, 28914 Leganés, Madrid, Spain; 3 Department of Translational Medical Sciences, University of Campania “Luigi Vanvitelli”, 80138 Naples, Italy; cecilia.calabrese@unicampania.it

**Keywords:** myotonic dystrophies, sleep disorders, respiratory failure, excessive daytime sleepiness, non-invasive ventilation

## Abstract

**Introduction:** Myotonic dystrophies (DM) are progressive genetic disorders with multisystemic involvement, particularly affecting the muscular, respiratory, and neuropsychological systems. Myotonic dystrophy type 1 (DM1), or Steinert’s disease, may lead to severe respiratory complications, including sleep-disordered breathing and hypercapnia, often requiring noninvasive ventilation to manage respiratory failure. However, adherence to NIV remains a major challenge, often influenced by cognitive and psychological factors such as apathy and depression. This study aims to investigate the presence of depression and SDB in patients with DM1 initiating NIV, and to evaluate factors influencing adherence to ventilatory support. **Materials and Methods:** We selected 13 adult patients (≥18 years) with diagnosis of Steinert’s disease with respiratory impairment who needed to start respiratory support. Dysphagia was assessed in all patients at baseline by a videofluoroscopic swallow study. Beck’s Depression Inventory II was administered for measuring the severity of depression. The Montreal Cognitive Assessment was used as a screening tool to detect signs of neurocognitive disorders. We evaluated adherence to NIV. **Results:** The study population presented with sleep-disordered breathing, as indicated by a median apnea–hypopnea index (AHI) of 24 events per hour (IQR: 14.2–34.5) and an oxygen desaturation index (ODI) of 25 events per hour (IQR: 18–33). Adherence to NIV was obtained in seven patients. No difference in baseline lung function was observed. Adherent subjects had moderate hypercapnia at baseline; pCO2 was 52 vs. 49 mmHg. Non-adherent patients showed a higher prevalence of depression with a median BDI-II score of 18 vs. 6 in adherent patients. The findings highlight that psychological factors, especially depression, play a crucial role in adherence to NIV. Interestingly, depression was not linked to initial respiratory measurements but showed a significant association with nocturnal oxygen desaturation. This suggests that relying solely on clinical and respiratory assessments may not be adequate to predict or improve treatment adherence. **Conclusions:** Incorporating psychological evaluations and addressing mental health issues, such as depression, are essential steps to enhance NIV compliance and overall DM1 patient outcomes. A multidisciplinary approach combining respiratory and psycho-emotional interventions is crucial for effective disease management.

## 1. Introduction

Myotonic dystrophies (DM) are autosomal dominant genetic disorders characterized by progressive and multisystemic involvement, including myopathy, myotonia, cardiomyopathy, endocrine abnormalities, and neuropsychological deficits [1].

The two main forms of myotonic dystrophy are type 1 (DM1) and type 2 (DM2), which exhibit overlapping clinical features but differ in terms of severity and prevalence. DM1, also known as Steinert’s disease, is more common and presents with a broader range of manifestations, including congenital, infantile, and adult-onset forms. In contrast, DM2 is often milder but may be underdiagnosed due to its subtler presentation [2]. DM1 and DM2 are both associated with significant variability in muscular and non-muscular symptoms, including myotonia, muscular dystrophy, cataracts, hypogonadism, frontal baldness, cardiac and respiratory complications, and neuropsychological deficits, though they differ in their clinical presentations.

DM1 often includes cardiac arrhythmias, respiratory muscle weakness, dysphagia, and chronic aspiration, which reduce life expectancy, particularly in early-onset cases, with sudden death frequently linked to nighttime hypoxemia and cardiac issues [2,3].

Muscular dystrophy affects all skeletal muscles. These also include the respiratory muscles, which constitute the respiratory pump, the role of which is to move air into the lung, therefore overcoming the elastic and resistive forces exerted by the chest wall and lungs. The impairment of respiratory muscles (which in muscular dystrophy become progressively weak and/or fatigued), combined with the high load against which the pump has developed its pressure, determine ventilatory failure. The elastic properties of the lung are altered in muscular dystrophy patients, with the lung becoming less distensible. The cause of the reduced distensibility of the lung in muscular dystrophy is still not known [4]. Different hypothesis have been proposed: incomplete maturation of lung tissue in case of congenital disease, micro- or macroatelectasis induced by hypoventilation, increase in alveolar surface tension, fibrosis induced by recurrent aspiration and parenchymal disease. Bulbar musculature can also be involved in muscular dystrophy patients. The direct consequences are impairment of swallowing, speech impediments and ensuing significant airway obstruction during inspiration [4,5].

With the progression of the disease and respiratory muscle involvement, muscular dystrophy patients are more prone to rapid shallow breathing (RSB), hypoventilation and thoracoabdominal patterns, and asynchronous movement between the ribcage and abdomen. RSB is the result of the combination of at least three factors: respiratory muscle weakness and vagal and nonvagal afferents [4,5]. Vagal afferents come from the lung; nonvagal from the stiffened chest wall and weak respiratory muscles. These afferents are responsible for shortening the inspiratory time, thereby truncating the tidal volume and making RSB increase. The consequences of RSB and chest wall distortion caused by muscle weakness are the onset of atelectasis, which worsen the already constricted lungs and also promote nocturnal hypoventilation. Another mechanism is increased respiratory effort, which fatigues the already compromised respiratory muscles. Finally, the onset of hypercapnia triggers the reflex that increases ventilation to access oxygen, further contributing to respiratory muscle fatigue [6].

Sleep disturbances are highly prevalent in DM and significantly impact morbidity and quality of life.

In DM1, a wide range of sleep disorders can develop, including various forms of sleep-disordered breathing (SDB), such as ataxic breathing, reported in rare cases. Secondary insomnia, circadian rhythm disruption, and daytime hypersomnolence are also common. Excessive daytime sleepiness (EDS), often unrelated to nocturnal sleep quality, is one of the earliest symptoms reported and may overshadow muscular complaints in early disease stages. Hypersomnia in DM1 is typically unrelated to nighttime sleep quality and may instead reflect underlying central dysfunction [7,8].

Polysomnographic studies frequently reveal abnormalities such as Rapid Eye Movement (REM) sleep dysregulation, sleep-onset REM episodes, and narcolepsy-like features. Disruptions in circadian rhythms may exacerbate daytime sleep-related symptoms [7,8].

While data on DM2 remain limited, studies have documented SDB, EDS, restless legs syndrome (RLS), and REM sleep disturbances [9]. Unlike DM1, SDB in DM2 predominantly involves obstructive respiratory events, with rare central apnea patterns. In nearly half of patients, mortality is due to respiratory failure caused by respiratory muscle weakness leading to hypercapnia.

In DM1, long-term noninvasive ventilation (NIV) using bilevel positive airway pressure (BiPAP) can relieve respiratory failure symptoms, normalize gas exchange, and improve life expectancy. However, adherence is often low due to reduced symptom perception, apathy, and cognitive impairment [10].

Adherence to NIV is presumed to be poor in patients with DM1, mainly due to cognitive and behavioral impairments such as apathy [11]. Apathy, defined as a marked reduction in motivation, initiative, and interest in daily activities, is a neuropsychological feature frequently observed in DM1. This condition is often underdiagnosed but may have significant clinical implications, limiting the patient’s active involvement in therapeutic management and reducing adherence to complex treatments like NIV. These manifestations are thought to stem from dysfunctions in fronto-subcortical circuits responsible for motivated behavior regulation [12]. Indeed, despite the existence of recommendations and clinical guidelines on when to initiate NIV, determining the optimal timing for starting ventilatory support and maintaining adherence remains a challenge in individual DM1 patients [13]. Moreover, current data on the psychological profiles of DM1 patients are limited, and it is still unclear to what extent depression or apathy may directly affect therapeutic compliance with NIV.

In this context, our study aims to investigate the presence of depression and SDB in patients with DM1 initiating NIV, and to evaluate factors influencing adherence to ventilatory support.

## 2. Materials and Methods

This is a single-center observational study including patients affected by MD and referred to the Respiratory Pathophysiology and Rehabilitation Unit of Monaldi Hospital in Naples, Italy, between 2023 and 2024. This study was performed in line with the principles of the Declaration of Helsinki. The study was approved by the local ethic committee. We included adult patients (≥18 years) with diagnosis of Steinert’s disease. All patients received a periodic multisystemic follow-up. Patients with a clinical history of progressive respiratory impairment requiring mechanical ventilation were enrolled. Pharmacological therapy varied based on subjective manifestations of the disease. None of the patients were receiving psychoactive medications at the time of assessment.

During the protocol all patients received a complete respiratory evaluation at baseline. We recorded lung function including the forced vital capacity (FVC), forced expiratory volume in the first second (FEV_1_), Maximum Inspiratory Pressure (MIP), Maximum Expiratory Pressure (MEP), and cough peak expiratory flow (CPEF), and also included blood gas analysis with pH, partial pressure of oxygen (pO_2_), and partial pressure of carbon dioxide (pCO_2_). Moreover, cardiorespiratory monitoring during nighttime was recorded at baseline; we recorded the Apnea Hypopnea Index (AHI), the Oxygen Desaturation Index (ODI) and the time under 90% of saturation of oxygen (SpO_2_). The Epworth sleepiness scale (ESS) was administered at baseline to estimate the impact of nocturnal respiratory failure on daytime performance. Finally, a psychometric test was included. Beck’s Depression Inventory II (BDI-II) is an easy intuitive self-administered assessment for measuring the severity of depression. It includes 21 items, relating to symptoms of depression such as hopelessness and irritability, cognitions such as guilt or feelings of being punished, as well as physical symptoms such as fatigue, weight loss, and lack of interest in sex. Each answer is scored on a scale with values of 0 to 3. Higher total scores indicate more severe depressive symptoms. The standardized cutoffs exclude depression between 0 and 13 points [14].

The Montreal Cognitive Assessment (MoCA) was used as a screening tool to detect signs of neurocognitive disorder as defined by the Statistical Manual for psychiatric Disorders 5 (DSM-5). It is broadly used across various clinical settings, including populations affected by neurological and neuromuscular disorders. The MoCA is a multidomain tool consisting of subtests that evaluate several cognitive domains, including attention and concentration, short-term memory, language, executive functions, visuospatial abilities, abstract thinking, calculation, and temporal-spatial orientation. The maximum total score is 30 points, with a conventional clinical cut-off of 26, below which cognitive impairment is suspected [15]. In the present study, we followed the recommendations of Carson et al. [16] and adopted a lower cut-off score of 23 to reduce the risk of false positives.

All enrolled patients underwent the Montreal Cognitive Assessment (MoCA) to screen for neurocognitive impairment. The median score was 23, using a diagnostic cut-off of 23 points to minimize false positives in neuromuscular disorders.

Dysphagia was assessed in all patients at baseline by a videofluoroscopic swallow study (VFSS). Adherence to NIV was considered to be at least 4 h per day in 5 days a week, based on the patient and caregiver declaration and verified by machine data download.

### Statistical Analysis

Data are presented as median and interquartile range (IQR) for continuous variables and as numbers (*n*) and percentages (%) for categorical variables; 95% confidence intervals (95% CI) were calculated. The normality of the data was assessed using the Anderson–Darling test. Differences between subgroups were evaluated using the unpaired Student’s *t*-test. The chi-square test was used to compare categorical variables. All statistical tests were two-tailed, and *p*-values < 0.05 were considered statistically significant. Linear regression was used to model the relationship between two continuous variables and estimate the value of a response. Statistical analyses were performed using GraphPad Prism version 9.1.0 (GraphPad Software, San Diego, CA, USA).

## 3. Results

Thirteen patients with DM1 were enrolled, nine males and four females. The median age was 48 years (40.5–54.5). The median BMI was 29 kg/m^2^ (24.5–32.5). Lung function was determined at baseline. The median FVC was 66% of expected value (57.5–70), the median FEV_1_ was 66% of expected value (1.89–2.55). All patients showed a restrictive ventilatory defect at diagnosis.

The most common acid–base imbalance was hypercapnia, with a median pCO_2_ of 50 mmHg (interquartile range [IQR]: 49–54) and a median pO_2_ of 67 mmHg (IQR: 61.5–75). The study population presented with sleep-disordered breathing, as indicated by a median apnea–hypopnea index (AHI) of 24 events per hour (IQR: 14.2–34.5) and an oxygen desaturation index (ODI) of 25 events per hour (IQR: 18–33). Additionally, 61.5% of patients showed an ineffective cough at the time of diagnosis. Baseline characteristics, respiratory parameters, associated conditions, and therapeutic interventions are summarized in Table 1.

After receiving a diagnosis of sleep disordered breathing, all subjects were prescribed appropriate therapy. Education about NIV was the main objective of the subsequent follow-up visit, as many patients experienced opposition to the mask. Twelve patients were treated with NIV, while one patient after 8 months, due to rapid clinical worsening, received invasive mechanical ventilation (IMV). The respiratory support was initiated prior to enrollment in this study (6–12 months). All patients were treated with NIV with personalized parameters, titrating pressure support, inspiratory and expiratory times, and end-expiratory pressure until respiratory events resolved. All patients tried a nasal interface, a nose–mouth interface, and sometimes a nasal pillow during the adaptation phase. Four patients used the full-face mask (two adherent to NIV and two non-adherent), seven patients used the nasal mask (four adherent and three non-adherent) and two patients used nasal pillows (one adherent and one non-adherent).

Adherence was obtained in seven patients, while six patients were inconstant. Adherent patients were younger; the median age was 42 years vs. 54.5. No difference in baseline lung function was observed. Adherent subjects had moderate hypercapnia at baseline, pCO_2_ was 52 vs. 49 mmHg, but non-adherent patients had more disordered sleep breathing with AHI 32 and ODI 33 per hour. In fact, non-adherent patients showed significant sleepiness at baseline, with ESS 17.5 vs. 15 in the adherent group. Remarkably, non-adherent patients showed a higher prevalence of depression with a median BDI-II score of 18 vs. 6 in adherent patients.

**Figure 1:**
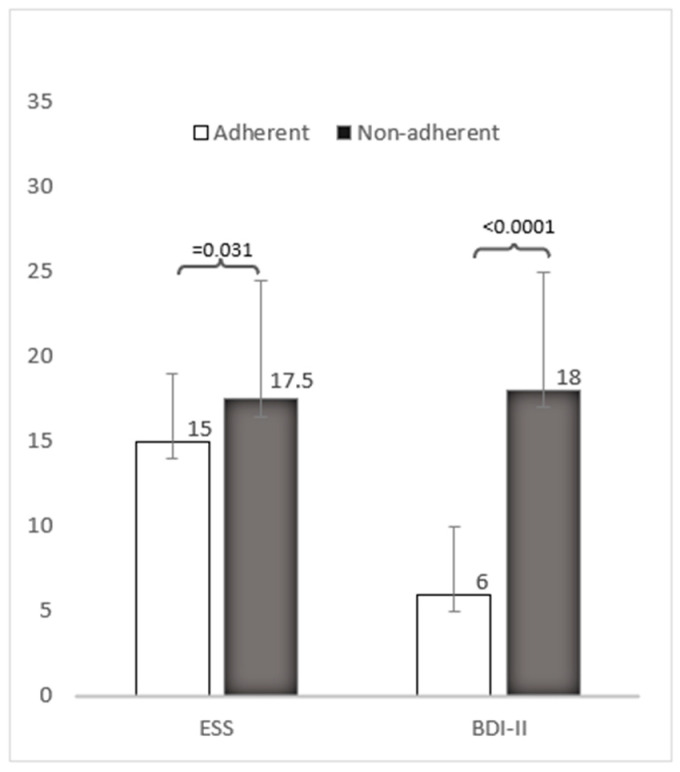
Epworth sleepiness scale (ESS) and Beck Depression Inventory 2 (BDI-II) in adherent versus non-adherent subgroups.

Moreover, five patients (38%) scored < 23, indicating mild impairment mainly in attention, short-term memory, and executive functions. Of these, four were non-adherent to NIV and had lower MoCA scores (median 21) than adherent patients (median 24), despite no significant differences in respiratory function parameters (FVC, FEV_1_). Differences between the adherent and non-adherent patients are reported in Table 2 and Figure 1.

**Figure 2:**
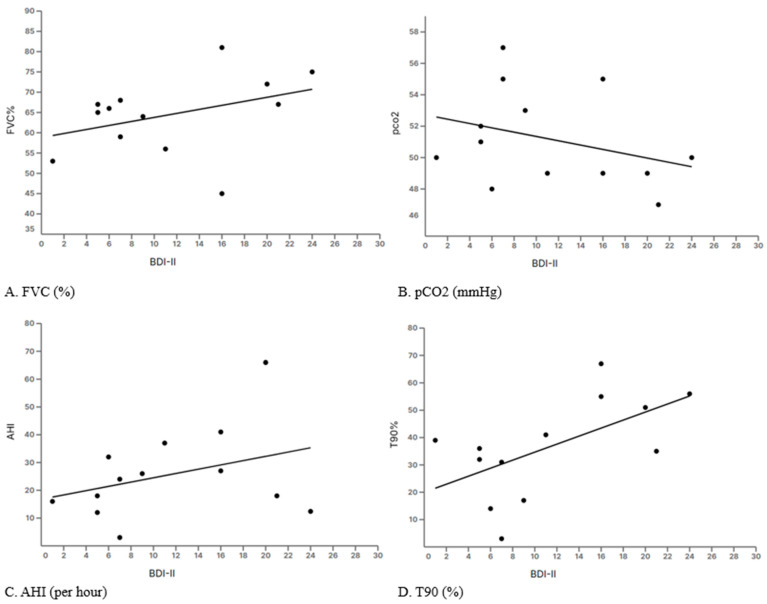
Linear regression between Beck’s Depression Inventory and lung function parameters. FVC% (**A**), pCO_2_ (**B**), AHI (**C**), T90 (**D**).

Linear regression analyses showed that depressive symptom severity, as measured by the BDI-II, was not significantly associated with baseline lung function parameters including FVC% (R^2^ = 0.1433, p = 0.20), pCO_2_ (R^2^ = 0.106, p = 0.28), or AHI (R^2^ = 0.12, p = 0.25) (Figure 2A–C). Notably, BDI-II scores were significantly correlated with baseline nocturnal hypoxemia, with higher T90% values predicting higher depression scores (R^2^ = 0.3436, p = 0.035*) (Figure 2D).

## 4. Discussion

This observational study provides valuable insights into the complex interplay between SDB, depression, and adherence to NIV in patients with DM1. The findings underscore several important clinical considerations that can inform management strategies for this complex patient population.

Our results offer additional data on respiratory support in a small cohort of DM1 patients eligible for NIV. Consistent with the literature, we confirm that respiratory failure is common among these patients and that adherence to NIV remains a significant challenge. Patients typically exhibit a restrictive ventilatory pattern, requiring regular comprehensive respiratory evaluations during follow-up to monitor disease progression and optimize therapeutic management [17].

The high prevalence of SDB in our cohort aligns with existing literature, which indicates that respiratory complications are frequent in DM1 due to muscle weakness and respiratory muscle impairment. The median AHI of 24/h reflects moderate to severe sleep apnea, which can significantly impact quality of life and exacerbate daytime fatigue and cognitive impairment. Notably, non-adherent patients exhibited more severe sleep disturbances with higher AHI and ESS scores, suggesting that the severity of sleepiness and sleep disruption may influence patient willingness or ability to adhere to NIV therapy.

Most patients in our study exhibited mild to moderate muscular involvement and maintained autonomy in walking and daily routines. However, these preserved functions did not always reflect their respiratory status during sleep. Ferrari Aggradi et al. observed that half of their patients had reduced forced vital capacity (FVC) at baseline and required nocturnal NIV, often related to older age at diagnosis, longer disease duration, and factors such as pathological BMI [18]. These findings underscore the importance of early detection and ongoing assessment of respiratory function, even in patients with relatively mild muscular symptoms.

Our data also reveal a strong association between depression and NIV adherence. Patients who were non-adherent demonstrated significantly higher median BDI-II scores (18 vs. 6), indicating more severe depressive symptoms. This correlation suggests that depression may serve as a barrier to consistent NIV use, possibly due to reduced motivation, apathy, or cognitive impairments common in DM1 [19]. Additionally, adherent patients tended to be younger (median age 42) compared to non-adherent ones (median 54.5), possibly reflecting greater adaptability or social support—factors that warrant further investigation.

These findings highlight the importance of routine psychological assessment and the integration of mental health support in the management of DM1 patients undergoing respiratory therapy.

Interestingly, lung function parameters such as FVC and FEV1 did not differ significantly between groups. However, sleep parameters, particularly T90 (the percentage of sleep time spent with oxygen saturation below 90%), were more compromised in non-adherent patients. This indicates that sleep quality and nocturnal hypoxemia might influence adherence, emphasizing the need for tailored interventions addressing both physiological and psychological factors to improve compliance. This study sheds light on the link between depressive symptoms, cognitive function and NIV adherence in Myotonic Dystrophy type 1 (DM1). Psychological profile appears to be an independent predictor of adherence. Notably, BDI-II scores were not significantly influenced by baseline respiratory and sleep metrics like FVC%, pCO_2_, AHI or ODI, but were significantly related to baseline T90. This suggests that psychological status can affect adherence independently of the severity of respiratory impairment.

Remarkably, cognitive decline was likewise unrelated to baseline respiratory parameters (FVC, FEV_1_, pCO_2_), suggesting a trajectory independent from respiratory function. However, lower MoCA scores were associated with depressive symptoms and NIV non-adherence, despite similar respiratory parameters suggesting a partially independent neuropsychological trajectory [18,19]. Collectively, the results indicate that cognitive impairment, together with depression, may predict poor NIV adherence in DM1 regardless of respiratory dysfunction severity, consistent with previous reports of executive dysfunction and apathy due to fronto-subcortical involvement [11,12].

DM1 is characterized by multisystemic involvement, including central nervous system manifestations. Cognitive changes, particularly executive dysfunction, apathy, and reduced information processing speed are frequently reported and are believed to result from structural and functional alterations in fronto-subcortical and white matter tracts [11,12]. Furthermore, the need for NIV often reflects more advanced disease stages, with greater muscular and respiratory compromise; this disease burden may further exacerbate cognitive and affective symptoms through mechanisms such as chronic hypoxemia, hypercapnia, sleep disruption, and increased psychological stress [6,7]. Our findings of lower MoCA scores and higher depression indices in NIV-dependent or non-adherent patients support this multidimensional vulnerability. These results underscore the importance of integrating neuropsychological and mental health support into the multidisciplinary care of DM1.

As NIV adherence is key to managing chronic respiratory failure in DM1, early detection of cognitive and affective changes could guide tailored interventions to improve compliance. These results support routine cognitive and psychological assessments in DM1 follow-up alongside respiratory and neuromuscular evaluations. MoCA and BDI-II screening may therefore be a useful component of multidisciplinary follow-up in DM1.

Caregiver support and education should also be adapted to each patient’s neuropsychological profile to address barriers to adherence.

The main limitation of this study is the small sample size, an inevitable consequence of investigating Steinert’s disease (DM1), a rare neuromuscular disorder with an overall prevalence generally estimated at around 3–15 cases per 100,000 individuals [1]. The limited number of participants reduces statistical power and generalizability; however, the available literature on mechanical ventilation and psychosocial outcomes in DM1 is mostly based on small observational cohorts, with heterogeneous methodologies and incomplete assessment of patient-reported outcomes [11,13]. Despite this, our study stands out for its methodological breadth.

In contrast to most previous reports, we applied a comprehensive assessment protocol, including full respiratory testing (spirometry, inspiratory/expiratory pressures, nocturnal cardiorespiratory monitoring, blood gases) alongside standardized psychological and cognitive measures (BDI-II, MoCA) and dysphagia screening [14,15]. This approach provides a more integrated view of both ventilatory and psychosocial dimensions in DM1.

From a clinical perspective, our insights advocate for a multidisciplinary approach in managing DM1 patients. Routine psychological assessments should be integrated into the care pathway, with particular attention to depressive symptoms, which can hinder effective NIV use. Interventions such as psychological counseling, personalized education, and support during NIV initiation—addressing issues like mask fitting opposition—are essential to foster adherence. The involvement of caregivers and tailored support strategies can further enhance compliance.

Future studies should address the inherent rarity of Steinert’s disease by promoting multicenter collaborations and larger prospective cohorts to confirm these findings and refine the identification of predictors for respiratory decline and NIV adherence. In addition, longitudinal follow-up and the systematic use of standardized patient-reported outcome measures would be crucial to better understand the influence of cognitive and psychological factors on clinical management and quality of life.

## 5. Conclusions

Respiratory management in DM1 requires a multidisciplinary approach that integrates the assessment and treatment of muscle weakness, respiratory impairment, and psychological factors such as depression and apathy. Early identification and management of mood and sleep disorders are essential to optimize adherence to NIV, reduce morbidity, and improve quality of life. In this context, routine psychological assessments should be incorporated into the clinical pathway, with particular attention to depressive symptoms and behavioral disturbances that may compromise therapeutic adherence. Targeted interventions such as psychological counselling, personalized education, and support during NIV initiation—including addressing specific issues like mask intolerance—can significantly enhance compliance. The involvement of caregivers and individualized support strategies are additional key elements to improve treatment success. Respiratory clinical evaluation alone is insufficient; integrating psychological assessments into follow-up allows for personalized interventions aimed at sustaining treatment continuity. Early psycho-emotional support and management of depressive symptoms can prevent treatment failure and improve overall clinical outcomes.

Future research with larger populations should focus on developing effective strategies to address these complex, interrelated aspects in an integrated manner to enhance the quality of care in patients with DM1.

## Figures and Tables

**Table 1:** Baseline clinical, respiratory, and therapeutic data. Bold font indicates the category groups of data.

Baseline	All (*n* = 13)
**General characteristics**	
Age, years, median (IQR)	48 (40.5–54.5)
Female, *n* (%)	4 (31)
BMI, kg/m^2^, median (IQR)	29 (24.5–32.5)
**Lung function**	
FVC, L, median (IQR)	2.63 (2.33–2.94)
FVC, % predicted, median (IQR)	66 (57.5–70)
FEV_1_, L, median (IQR)	2.29 (1.89–2.55)
FEV_1_, % predicted, median (IQR)	66 (55–73.5)
**Blood gas analysis**	
pH, median (IQR)	7.39 (7.36–7.41)
pCO_2_, mmHg, median (IQR)	50 (49–54)
pO_2_, mmHg, median (IQR)	67 (61.5–75)
**Respiratory function during sleep**	
AHI, per hour, median (IQR)	24 (14.2–34.5)
ODI, per hour, median (IQR)	25 (18–33)
T90, %, median (IQR)	36 (24–53)
ESS, median (IQR)	15 (11.5–17.5)
**Associated conditions**	
Ineffective cough, *n* (%)	8 (61.5)
Dysphagia, *n* (%)	3 (23)
BDI-II, median (IQR)	9 (5.5–18)
Depression [BDI > 13], *n* (%)	5 (38.5)
**Therapy**	
IMV, *n* (%)	1 (8)
NIV, *n* (%)	12 (92)
Cough assist, *n* (%)	8 (61.5)
**Caregiver**, *n* (%)	9 (69.2)
**Adherence to NIV**, *n* (%)	7 (53.8)

BMI: body mass index. kg/m^2^: kilogram per square meter. FVC: forced vital capacity. L: liter. FEV_1_: forced expiratory volume in the first second. pCO_2_: partial pressure of carbon dioxide. pO_2_: partial pressure of oxygen. AHI: Apnea–Hypopnea Index. ODI: Oxygen Desaturation Index. T90: time under 90% of SpO_2_. ESS: Epworth sleepiness scale. BDI-II: Beck’s Depression Inventory II. IMV: invasive mechanical ventilation.

**Table 2:** Comparison of baseline characteristics between adherent (Yes) and non-adherent (No) patients.

Adherence	All (*n* = 13)	Yes (*n* = 7)	No (*n* = 6)	*p*-Value
Age, years, median (IQR)	48 (40.5–54.5)	42 (38–48)	54.5 (48–64)	0.016 *
Female, *n* (%)	4 (31)	3 (43)	1 (17)	0.55
FVC, % predicted, median (IQR)	66.5 (62–67.5)	65 (59–67)	69.5 (56–75)	0.061
FEV_1_, % predicted, median (IQR)	66 (55–73.5)	66 (55–70)	70 (55–75)	0.67
pCO_2_, mmHg, median (IQR)	50 (49–54)	52 (50–55)	49 (49–50)	0.15
pO_2_, mmHg, median (IQR)	67 (61.5–75)	69 (63–74)	64 (61–76)	0.074
AHI, per hour, median (IQR)	24 (14.2–34.5)	18 (12–26)	32 (18–41)	0.098
ODI, per hour, median (IQR)	25 (18–33)	20 (18–27)	33 (23–44)	0.075
T90, %, median (IQR)	35.5 (17–51)	24 (8.5–34)	53 (41–56)	0.0031 *
ESS, median (IQR)	15 (11.5–17.5)	15 (12–16)	17.5 (11–18)	0.031 *
BDI-II, median (IQR)	9 (5.5–18)	6 (5–7)	18 (16–21)	0.0001 *
MoCA, median (IQR)	23 (22–25)	25 (24–26)	22 (21–22)	0.0013 *
Caregiver	9 (69.2)	6 (86)	3 (50)	0.26
NIV interfaces: n (%)				
-Full face	4 (31)	2 (29)	2 (33)	
-Nasal	7 (54)	4 (57)	3 (50)	
-Nasal pillow	2 (15)	1 (14)	1 (17)	

FVC: forced vital capacity. L: liter. FEV1: forced expiratory volume in the first second. pCO_2_: partial pressure of carbon dioxide. pO_2_: partial pressure of oxygen. AHI: Apnea–Hypopnea Index. ODI: Oxygen Desaturation Index. T90: time under 90% of SpO_2_. ESS: Epworth sleepiness scale. BDI-II: Beck’s Depression Inventory II. MoCA: Montreal Cognitive Assessment. “*” indicates statistically significant results with *p*-value < 0.05.

## Data Availability

The data that support the findings of this study are available from the corresponding author upon reasonable request, in order to preserve participant confidentiality.
